# Supplementary material for: Phenolic Compounds of Rose Hips of Some Rosa Species and Their Hybrids Native Grown in the South-West of Slovenia during a Two-Year Period (2020–2021)
Source: Foods. 2023 May 11;12(10):1952. doi: 10.3390/foods12101952 (PMC10217035; doi:10.3390/foods12101952)
Supplement: Supplementary file 1 [file foods-12-01952-s001.zip › foods-2349152-supplementary.pdf]

## Supplementary Material

**Table S1.** The content ± standard error (mg/kg FW) of phenolic compounds (HBA, HCA, gallotannins and ellagitannins) of the rosehips of *R.* × *R. glauca* and *R. corymbifera* over a period of two years (2020-2021) and *R. gallica* and *R. subcanina*) in 2021. Different lowercase letters indicate statistically significant differences between species in each year separated into flesh with skin and seed.

| Phenolic Group | Compound | | ***R. x R. glauca*** | | ***R. corymbifera*** | | ***R. subcanina*** | | ***R. gallica*** | |
|---|---|---|---|---|---|---|---|---|---|---|
| | | | **Flesh and Skin** | **Seeds** | **Flesh and skin** | **Seeds** | **Flesh and skin** | **Seeds** | **Flesh and skin** | **Seeds** |
| hydroxybenzoic acid derivatives (HBA) | gallic acid | 2021 | 7.64 ± 1.89 a | 103.01 ± 59.62 c | - | 30.02 ± 19.92 a | - | 67.03 ± 25.93 b | 62.30 ± 28.02 b | - |
| | | 2020 | 25.92 ± 11.61 | 77.06 ± 56.02 b | - | 14.02 ± 6.98 a | - | - | - | - |
| | galloyl quinic acid | 2021 | 57.34 ± 13.62 | 0.68 ± 0.31 | - | - | - | - | - | - |
| | | | 178.90 ± 84.52 | 9.92 ± 7.61 | - | - | - | - | - | - |
| | ellagic acid pentoside 1 | 2021 | 4.61 ± 3.82 c | 3.92 ± 3.04 b | 25.52 ± 1.52 b | 7.65 ± 5.03 c | 1.32 ± 0.63 a | 3.32 ± 1.41 a | 31.95 ± 1.06 b | 2.95 ± 1.03 a |
| | | 2020 | 11.52 ± 4.63 b | 7.62 ± 2.93 a | 7.03 ± 3.95 a | 159.30 ± 60.38 b | - | - | - | - |
| | ellagic acid pentoside 2 | 2021 | 0.71 ± 0.68 a | 2.51 ± 3.52 c | 8.92 ± 5.92 c | 2.03 ± 1.68 b | 3.01 ± 0.81 b | 2.01 ± 0.63 b | 48.02 ± 11.02 d | 0.81 ± 0.62 a |
| | | 2020 | 5.71 ± 2.01 a | 0.09 ± 0.01 | 29.83 ± 14.51 b | - | - | - | - | - |
| | taxifolin pentoside 1 | 2021 | - | - | 0.36 ± 0.18 b | - | 1.06 ± 0.60 a | - | - | - |
| | | 2020 | - | - | 0.73 ± 0.47 | - | - | - | - | - |
| | taxifolin pentoside 2 | 2021 | - | - | 1.56 ± 1.03 b | - | 0.61 ± 0.21 a | - | - | - |
| | | 2020 | - | - | 4.90 ± 3.01 | - | - | - | - | - |
| | taxifolin pentoside 3 | 2021 | - | - | 7.29 ± 4.92 a | - | 6.80 ± 4.03 a | - | - | - |
| | | 2020 | - | - | 20.01 ± 8.62 | - | - | - | - | - |
| | ellagic acid rhamnoside | 2021 | - | - | - | - | - | 0.32 ± 0.09 | - | - |
| | | 2020 | - | - | - | - | - | - | - | - |
| | methyl ellagic acid pentoside 1 | 2021 | - | 2.98 ± 0.51 | - | - | - | - | - | - |
| | | 2020 | - | 5.92 ± 4.05 b | - | 0.24 ± 0.12 a | - | - | - | - |
| | methyl ellagic acid pentoside 2 | 2021 | - | 0.81 ± 0.39 | - | - | - | - | - | - |
| | | 2020 | - | 2.87 ± 2.61 | - | - | - | - | - | - |
| | **TOTAL** | **2021** | **70.30 ± 20.01 c** | **113.91 ± 67.39 d** | **43.65 ± 13.57 b** | **39.7 ± 26.63 b** | **12.80 ± 6.28 a** | **72.68 ± 28.06 c** | **142.27 ± 40.10 d** | **3.76 ± 1.65 a** |
| | | **2020** | **222.05 ± 102.77 b** | **103.48 ± 73.23 a** | **62.50 ± 30.56 a** | **173.56 ± 67.48 a** | **-** | - | - | - |
| hydroxycinnamic acid | *p*-coumaric acid hexoside 1 | 2021 | 3.02 ± 1.91 b | 3.02 ± 0.22 c | 11.09 ± 3.09 a | 0.22 ± 0.03 a | 12.03 ± 8.31 a | 0.22 ± 0.09 a | 2.98 ± 0.62 ab | 0.55 ± 0.21 b |
| | | 2020 | 4.03 ± 2.02 a | 0.61 ± 0.42 a | 39.01 ± 20.03 b | 0.19 ± 0.06 b | - | - | - | - |

| | | | | | | | | | | |
|---|---|---|---|---|---|---|---|---|---|---|
| | *p*-coumaric acid hexoside 2 | 2021 | 8.65 ± 3.05 b | - | 3.15 ± 0.98 a | - | 3.22 ± 2.64 a | - | - | - |
| | | 2020 | 16.51 ± 4.98 a | - | 16.58 ± 9.01 a | - | - | - | - | - |
| | 5-caffeoylquinic acid 1 | 2021 | 7.92 ± 2.97 a | - | 40.05 ± 19.52 b | 1.05 ± 0.82 b | 30.01 ± 20.03 b | 309.95 ± 97.33 c | 95.99 ± 12.01 c | 0.32 ± 0.11 a |
| | | 2020 | 8.52 ± 3.02 a | - | 17.33 ± 5.56 b | 1.68 ± 0.92 | - | - | - | - |
| | sinapic acid hexoside 1 | 2021 | 15.01 ± 6.10 b | 88.91 ± 32.05 b | 64.92 ± 48.36 c | 1.19 ± 0.71 a | 119.95 ± 70.66 d | 1.31 ± 0.58 a | 38.02 ± 20.33 a | - |
| | | 2020 | 20.09 ± 10.10 a | 37.52 ± 28.03 b | 309.02 ± 140.02 b | 1.05 ± 0.40 a | - | - | - | - |
| | sinapic acid hexoside 2 | 2021 | 23.36 ± 10.35 b | - | 20.03 ± 10.05 b | - | 20.03 ± 10.24 b | - | 1.96 ± 0.75 a | - |
| | | 2020 | 30.92 ± 14.09 b | - | 210.18 ± 140.52 a | - | - | - | - | - |
| | 5-caffeoylquinic acid 2 | 2021 | 2.95 ± 1.05 a | - | 20.01 ± 6.33 c | - | 10.11 ± 8.35 b | 0.22 ± 0.11 a | 0.26 ± 0.11 a | 0.39 ± 0.03 b |
| | | 2020 | 4.01 ± 1.02 b | - | 30.05 ± 11.98 a | - | - | - | - | - |
| | 5-*p*-coumaroylquinic acid 1 | 2021 | 1.02 ± 0.92 a | 4.03 ± 3.01 c | 10.58 ± 6.09 d | 1.33 ± 0.92 b | 2.65 ± 1.69 b | 2.97 ± 1.04 c | 8.01 ± 2.35 c | 0.10 ± 0.05 a |
| | | 2020 | 1.05 ± 0.98 a | 9.42 ± 1.09 b | 115.98 ± 76.53 b | 1.12 ± 1.03 a | - | - | - | - |
| | 5-*p*-coumaroylquinic acid 2 | 2021 | 0.41 ± 0.15 b | - | 0.21 ± 0.09 a | - | 0.19 ± 0.08 a | 0.66 ± 0.62 b | - | 0.22 ± 0.04 a |
| | | 2020 | 3.05 ± 1.65 b | - | 0.91 ± 0.51 | 0.15 ± 0.02 a | - | - | - | - |
| | sinapic acid hexoside 3 | 2021 | 2.91 ± 1.03 | - | - | - | - | - | - | - |
| | | 2020 | 3.01 ± 4.93 | - | - | - | - | - | - | - |
| | 3-*p*-coumaroylquinic acid | 2021 | 1.05 ± 1.01 a | 1.02 ± 0.45 a | 1.75 ± 0.21 a | 53.82 ± 23.85 b | 4.11 ± 2.65 b | 12.75 ± 2.49 a | - | - |
| | | 2020 | 4.21 ± 1.08 a | 1.02 ± 0.62 a | 30.10 ± 9.02 b | 50.22 ± 16.03 b | - | - | - | - |
| | 3-feruloyquinic acid | 2021 | 1.01 ± 0.82 | 1.99 ± 0.89 | - | - | - | - | - | - |
| | | 2020 | 7.93 ± 5.03 | 15.03 ± 1.56 | - | - | - | - | - | - |
| | trigalloyl quinic acid 1 | 2021 | - | 37.03 ± 20.05 b | - | - | - | 0.51 ± 0.21 a | - | - |
| | | 2020 | - | 16.28 ± 7.03 | - | - | - | - | - | - |
| | trigalloyl quinic acid 2 | 2021 | - | - | - | - | - | 0.10 ± 0.08 | - | - |
| | | 2020 | - | - | - | - | - | - | - | - |
| | **TOTAL** | **2021** | **67.31 ± 29.36 a** | **136.00 ± 56.67 c** | **171.79 ± 94.72 b** | **57.61 ± 26.33 b** | **202.30 ± 124.65 b** | **328.69 ± 102.55 d** | **147.22 ± 36.17 b** | **1.58 ± 0.44 a** |
| | | **2020** | **103.33 ± 48.9 b** | **79.88 ± 38.75 b** | **769.16 ± 413.18 a** | **54.41 ± 18.46 a** | **-** | **-** | **-** | **-** |
| gallotannins | digalloyl hexoside 1 | 2021 | 77.92 ± 53.01 b | 48.27 ± 15.33 c | 112.35 ± 62.42 b | 36.42 ± 16.02 b | 206.02 ± 92.50 c | 103.02 ± 68.33 a | 39.50 ± 24.03 a | 41.02 ± 19.05 bc |
| | | 2020 | 273.45 ± 109.02 a | 1.05 ± 0.41 b | 362.05 ± 154.32 b | 3.01 ± 1.98 a | - | - | - | - |
| | digalloyl hexoside 2 | 2021 | 82.05 ± 51.02 c | 0.31 ± 0.21 b | 6.08 ± 2.08 a | 0.15 ± 0.03 a | 52.33 ± 19.98 b | 42.55 ± 9.01 c | 151.42 ± 69.05 d | 58.15 ± 34.21 d |
| | | 2020 | 146.02 ± 92.10 b | 0.42 ± 0.22 a | 71.44 ± 42.05 a | 13.29 ± 11.30 b | - | - | - | - |
| | methyl gallate hexoside | 2021 | 62.34 ± 37.99 b | 52.03 ± 41.36 a | 50.22 ± 17.93 a | 77.36 ± 42.05 b | 441.08 ± 221.05 d | 72.01 ± 58.32 b | 176.03 ± 45.11 c | 99.87 ± 29.05 c |
| | | 2020 | 112.05 ± 74.29 a | 86.33 ± 48.36 b | 612.03 ± 311.20 b | 13.05 ± 8.02 a | - | - | - | - |

| | | | | | | | | | | |
|---|---|---|---|---|---|---|---|---|---|---|
| | digalloyl quinic acid 1 | 2021 | 152.01 ± 78.14 b | 4.92 ± 2.62 a | 86.34 ± 43.55 a | 12.05 ± 5.01 b | 212.01 ± 123.05 b | 43.90 ± 34.90 c | 389.02 ± 121.03 c | - |
| | | 2020 | 142.91 ± 74.03 a | 27.92 ± 25.01 b | 341.52 ± 150 b | 3.85 ± 2.91 a | - | - | - | - |
| | digalloyl quinic acid 2 | 2021 | - | 0.68 ± 0.31 | - | - | - | - | - | - |
| | | 2020 | - | 14.58 ± 8.33 | - | - | - | - | - | - |
| | digalloyl pentoside | 2021 | 3.04 ± 1.05 | - | - | - | - | - | - | - |
| | | 2020 | 6.87 ± 3.02 | - | - | - | - | - | - | - |
| | trigalloy hexoside 1 | 2021 | - | 0.62 ± 0.22 a | - | 8.41 ± 6.64 b | - | 19.05 ± 0.17 c | - | 22.34 ± 13.44 c |
| | | 2020 | - | 1.60 ± 2.25 a | - | 12.01 ± 5.44 b | - | - | - | - |
| | trigalloy hexoside 2 | 2021 | - | 79.03 ± 39.05 b | - | - | - | - | - | 0.61 ± 0.15 a |
| | | 2020 | - | 42.55 ± 27.08 | - | - | - | - | - | - |
| | trigalloy hexoside 3 | 2021 | - | 81.54 ± 21.44 b | - | - | - | 2.41 ± 0.20 a | - | - |
| | | 2020 | - | 84.21 ± 41.36 | - | - | - | - | - | - |
| | methyl gallate rutinoside | 2021 | - | 3.20 ± 1.03 | 6.62 ± 3.82 b | - | 1.52 ± 0.33 a | - | 5.51 ± 3.52 b | - |
| | | 2020 | - | 11.36 ± 9.22 | 9.05 ± 7.52 | - | - | - | - | - |
| | methyl gallate acetyl dihexoside | 2021 | - | - | - | 7.01 ± 6.02 | - | - | - | - |
| | | 2020 | - | - | - | 0.49 ± 0.18 | - | - | - | - |
| | **TOTAL** | **2021** | **377.36 ± 221.21 b** | **270.60 ± 121.57 b** | **261.61 ± 129.8 a** | **141.40 ± 75.77 a** | **912.96 ± 456.91 d** | **282.94 ± 188.93 c** | **761.48 ± 262.74 c** | **221.99 ± 95.90 b** |
| | | **2020** | **681.30 ± 352.46 b** | **270.02 ± 162.24 b** | **1396.09 ± 515.09 a** | **45.70 ± 29.83 a** | **-** | **-** | **-** | **-** |
| ellagitannins | diHHDP hexoside 1 | 2021 | 141.03 ± 52.04 b | 57.02 ± 70.55 b | 89.62 ± 29.02 a | 91.04 ± 19.33 a | 380.02 ± 121.05 c | 69.55 ± 25.07 c | - | 59.02 ± 41.03 b |
| | | 2020 | 103.02 ± 75.32 a | 101.24 ± 39.08 b | 349.58 ± 190.24 b | 102.04 ± 50.43 a | - | - | - | - |
| | diHHDP hexoside 2 | 2021 | 23.15 ± 19.10 a | 0.44 ± 0.14 b | 41.03 ± 17.02 b | 7.02 ± 3.04 d | 31.42 ± 19.84 ab | 0.19 ± 0.05 a | - | 2.06 ± 0.87 c |
| | | 2020 | 24.94 ± 14.09 a | 3.01 ± 1.52 b | 49.02 ± 30.05 b | 2.05 ± 1.92 a | - | - | - | - |
| | digalloyl HHDP hexoside 3 | 2021 | - | - | - | - | - | 0.54 ± 0.21 | - | - |
| | | 2020 | - | - | - | - | - | - | - | - |
| | HHDP digalloyl hexoside isomer 1 | 2021 | 43.09 ± 32.44 b | 0.58 ± 0.32 a | 37.02 ± 7.55 b | - | 16.08 ± 6.02 a | - | 59.12 ± 1.80 c | 8.70 ± 4.59 b |
| | | 2020 | 54.55 ± 33.11 b | 0.89 ± 0.51 | 138.01 ± 71.22 a | - | - | - | - | - |
| | HHDP digalloyl hexoside isomer 2 | 2021 | 253.11 ± 69.01 c | 112.03 ± 67.45 b | 171.05 ± 52.08 bc | - | 49.32 ± 34.92 a | - | 144.33 ± 63.52 b | 2.04 ± 1.03 a |
| | | 2020 | 57.02 ± 29.08 a | 44.01 ± 29.84 | 242.05 ± 90.04 b | - | - | - | - | - |

| | | | | | | | | | |
|---|---|---|---|---|---|---|---|---|---|
| HHDP digalloyl hexoside isomer 3 | 2021 | 12.68 ± 4.22 a | - | 13.02 ± 6.44 a | - | 11.03 ± 7.01 a | - | 43.95 ± 23.05 b | - |
| | 2020 | 104.95 ± 60.52 a | - | 87.01 ± 59.62 b | - | - | - | - | - |
| galloyl bis HHDP hexoside 1 | 2021 | 5.03 ± 1.99 c | - | 3.44 ± 1.09 b | - | 3.12 ± 1.94 b | 1.01 ± 0.41 b | 0.11 ± 0.06 a | 0.21 ± 0.11 a |
| | 2020 | 6.33 ± 2.50 a | - | 18.23 ± 11.03 b | - | - | - | - | - |
| galloyl bis HHDP hexoside 2 | 2021 | 253.44 ± 68.95 c | 21.02 ± 12.05 c | 7.28 ± 2.55 a | - | 7.46 ± 3.02 a | 0.53 ± 0.39 a | 32.04 ± 12.25 b | 1.05 ± 0.45 b |
| | 2020 | 274.02 ± 92.03 a | - | 29.44 ± 14.35 b | - | - | - | - | - |
| HHDP galloyl hexoside 1 | 2021 | 5.01 ± 3.02 b | 4.02 ± 1.92 c | 4.05 ± 1.03 a | 14.55 ± 12.05 b | 49.95 ± 29.81 c | 0.72 ± 0.56 a | 64.05 ± 14.36 d | 3.02 ± 1.98 c |
| | 2020 | 13.90 ± 5.04 b | 1.99 ± 1.02 a | 7.21 ± 2.40 a | 22.34 ± 17.03 b | - | - | - | - |
| HHDP galloyl hexoside 2 | 2021 | - | 0.39 ± 0.09 | - | - | - | 0.44 ± 0.22 | - | - |
| | 2020 | - | 0.20 ± 0.08 | **-** | **-** | **-** | **-** | **-** | **-** |
| **TOTAL** | **2021** | **727.54 ± 241.77 c** | **213.50 ± 152.53 b** | **366.51 ± 136.78 a** | **112.61 ± 34.42 c** | **503.40 ± 223.61 b** | **72.98 ± 26.91 a** | **343.60 ± 115.04 a** | **76.10 ± 49.97 a** |
| | **2020** | **638.73 ± 248.69 a** | **151.34 ± 72.05 a** | **911.55 ± 468.95 b** | **126.43 ± 69.38 b** | **-** | **-** | **-** | **-** |

Note: (-) Compound was not detected.

**Table S2.** Contents ± standard error (mg/kg FW) of flavanols, flavonols, flavones and dihydrochalcone of 4 analyzed rosehips (*R.* × *R. glauca*, *R. corymbifera*, *R. gallica* and *R. subcanina*) over a period of two years (2020-2021). Different lowercase letters indicate statistically significant differences between species in each year separated to flesh with skin and seeds.

| Phenolic Group | Compound | | ***R.* × *R. glauca*** Flesh and skin | Seeds | ***R. corymbifera*** Flesh and skin | Seeds | ***R. subcanina*** Flesh and skin | Seeds | ***R. gallica*** Flesh and skin | Seeds |
|---|---|---|---|---|---|---|---|---|---|---|
| flavanols | procyanidin dimer 1 | 2021 | 1199.05 ± 959.04 c | 669.02 ± 594.03 b | 669.31 ± 280.35 b | 179.63 ± 90.03 a | 429.83 ± 251.04 a | 183.52 ± 145.03 a | 1853.44 ± 549.63 d | 149.33 ± 59.31 a |
| | | 2020 | 1179.03 ± 621.05 b | 99.52 ± 20.06 a | 841.09 ± 569.53 a | 99.81 ± 33.02 a | - | - | - | - |
| | procyanidin dimer 2 | 2021 | 511.02 ± 377.12 a | 2.04 ± 0.61 a | 685.04 ± 509.66 b | 7.05 ± 4.31 c | 1031.40 ± 620.03 c | 3.64 ± 1.99 b | 690.03 ± 161.04 b | - |
| | | 2020 | 761.05 ± 385.20 a | 3.01 ± 1.95 b | 1503.02 ± 642.03 b | 0.41 ± 0.19 a | - | - | - | - |
| | catechin | 2021 | 241.98 ± 117.04 b | 156.16 ± 40.52 c | 116.03 ± 110.20 a | 199.83 ± 100.28 c | 279.06 ± 150.33 b | 62.70 ± 16.53 b | 229.85 ± 109.32 b | 6.29 ± 3.92 a |
| | | 2020 | 612.31 ± 227.49 a | 99.82 ± 60.44 a | 191.62 ± 660.25 b | 170.03 ± 123.44 b | - | - | - | - |
| | catechin hexoside | 2021 | - | 120.04 ± 70.14 b | - | - | - | 0.18 ± 0.06 a | - | 124.03 ± 51.46 b |
| | | 2020 | - | 179.04 ± 70.02 | - | - | - | - | - | - |
| | procyanidin trimer 1 | 2021 | 242.05 ± 117.12 b | 590.10 ± 390.52 d | 200.02 ± 170.95 b | 1.04 ± 0.64 a | 40.53 ± 19.43 a | 180.53 ± 99.84 c | 287.65 ± 129.53 c | 2.39 ± 1.03 b |
| | | 2020 | 612.90 ± 227.01 a | 230.01 ± 110.54 | 2990.31 ± 1030.10 b | - | - | - | - | - |
| | procyanidin trimer 2 | 2021 | 289.12 ± 119.05 bc | - | 250.13 ± 139.06 b | 56.23 ± 24.35 ab | 319.25 ± 23.99 c | 0.59 ± 0.19 a | 1389.05 ± 305.62 d | 0.51 ± 0.36 a |
| | | 2020 | 404.21 ± 196.52 b | - | 96.04 ± 28.46 a | 36.24 ± 14.03 | - | - | - | - |
| | procyanidin dimer 3 | 2021 | 327.02 ± 143.95 a | - | 309.8 ± 110.30 a | 46.22 ± 19.08 c | 550.02 ± 182.65 b | 1.70 ± 1.47 a | 1543.02 ± 450.30 c | 3.38 ± 1.47 b |
| | | 2020 | 449.05 ± 196.04 b | - | 970.02 ± 621.05 | 48.01 ± 4.98 a | - | - | - | - |
| | epicatechin | 2021 | 6.03 ± 3.12 a | 28.44 ± 5.87 b | 46.99 ± 21.04 b | 30.05 ± 17.22 b | 39.64 ± 29.44 b | 0.49 ± 0.36 a | 329.67 ± 231.44 c | - |
| | | 2020 | 12.59 ± 7.02 a | 30.05 ± 29.01 a | 16.03 ± 8.97 a | 39.51 ± 12.03 b | - | - | - | - |
| | procyanidin dimer 4 | 2021 | 83.44 ± 41.06 c | 15.30 ± 11.03 b | 10.11 ± 6.08 b | 60.33 ± 41.05 c | 5.62 ± 2.95 a | 7.63 ± 0.48 a | 110.35 ± 29.87 c | - |
| | | 2020 | 174.03 ± 88.43 b | 29.84 ± 12.56 a | 1020.03 ± 469.92 a | 49.66 ± 29.91 b | - | - | - | - |
| | procyanidin trimer 3 | 2021 | 237.91 ± 116.23 a | - | - | 93.21 ± 49.83 c | - | 1.19 ± 1.26 a | 3568.42 ± 452.03 b | 3.33 ± 1.59 b |
| | | 2020 | 439.11 ± 157.21 | - | - | 97.02 ± 4.99 | - | - | - | - |
| | procyanidin dimer 5 | 2021 | 275.04 ± 125.06 b | - | 40.20 ± 15.02 a | 55.04 ± 29.64 b | - | 40.32 ± 26.05 a | 320.33 ± 130.62 b | - |
| | | 2020 | 291.08 ± 146.05 b | - | 230.05 ± 140.02 | 75.13 ± 32.05 a | - | - | - | - |
| | procyanidin dimer 6 | 2021 | - | - | - | - | - | - | 309.21 ± 144.53 | - |
| | | 2020 | - | - | - | - | - | - | - | - |
| | procyanidin trimer 4 | 2021 | - | 2.64 ± 0.54 a | - | 38.02 ± 29.05 c | - | 2 ± 0.64 a | - | 4.09 ± 2.11 b |
| | | 2020 | - | 1.40 ± 0.86 a | - | 14.62 ± 9.03 b | - | - | - | - |
| | procyanidin trimer 5 | 2021 | - | 22.05 ± 9.98 | - | - | - | - | 331.65 ± 95.86 | - |
| | | 2020 | - | 66.08 ± 24.55 | - | - | - | - | - | - |
| | procyanidin trimer 6 | 2021 | - | 12.68 ± 7.20 a | - | 69.83 ± 49.83 b | - | - | - | - |
| | | 2020 | - | 5.35 ± 2.03 a | - | 70.03 ± 59.10 b | - | - | - | - |

| | | | | | | | | | |
|---|---|---|---|---|---|---|---|---|---|
| procyanidin trimer 7 | 2021 | - | 49.97 ± 20.04 a | - | 100.04 ± 95.52 b | - | - | - | - |
| | 2020 | - | 19.01 ± 1.07 a | - | 99.65 ± 66.54 b | - | - | - | - |
| procyanidin trimer 10 | 2021 | - | 37.02 ± 20.55 | - | - | - | - | - | - |
| | 2020 | - | 43.07 ± 20.05 | - | - | - | - | - | - |
| procyanidin tetramer 1 | 2021 | - | - | - | - | - | - | 689.04 ± 159.62 | - |
| | 2020 | - | - | - | - | - | - | - | - |
| procyanidin tetramer 2 | 2021 | - | - | - | - | - | - | 108.25 ± 20.34 | - |
| | 2020 | - | - | - | - | - | - | - | - |
| procyanidin tetramer 3 | 2021 | - | - | - | - | - | - | 17.49 ± 4.99 | - |
| | 2020 | - | - | - | - | - | - | - | - |
| procyanidin tetramer 5 | 2021 | - | - | - | - | - | - | 152.63 ± 82.03 | - |
| | 2020 | - | - | - | - | - | - | - | - |
| PA dimer diglycoside | 2021 | 155.03 ± 32.05 ab | 34.15 ± 19.02 | 89.06 ± 39.16 a | - | 250.04 ± 71.43 b | - | - | - |
| | 2020 | 284.53 ± 146.02 a | 170.02 ± 80.40 | 522.66 ± 259.62 b | - | - | - | - | - |
| dimer PA monogallate 1 | 2021 | 92.10 ± 45.08 c | 14.93 ± 10.36 b | 30.10 ± 19.85 a | - | 53.62 ± 29.06 ab | - | 59.46 ± 16.41 b | 0.71 ± 0.42 a |
| | 2020 | 193.06 ± 96.62 a | 129.83 ± 75.01 | 249.03 ± 150.01 b | - | - | - | - | - |
| dimer PA monogallate 2 | 2021 | 126.04 ± 45.92 c | 0.19 ± 0.08 a | 22.45 ± 10.14 a | 7.11 ± 4.01 b | 25.43 ± 13.66 | - | 46.08 ± 31.96 b | - |
| | 2020 | 135.44 ± 67.08 a | 0.20 ± 0.09 a | 150.36 ± 99.83 a | 10.53 ± 2.85 b | - | - | - | - |
| dimer PA monogallate 3 | 2021 | - | 0.30 ± 0.20 a | - | - | - | - | 0.19 ± 0.03 | 0.54 ± 0.39 b |
| | 2020 | - | 0.21 ± 0.03 | - | - | - | - | - | - |
| PA dimer monoglycoside 1 | 2021 | - | - | 105.06 ± 50.88 b | 30.02 ± 17.22 b | 570.30 ± 20.85 c | 0.33 ± 0.18 a | 11.32 ± 4.93 a | - |
| | 2020 | - | - | 492.23 ± 219.74 | 2.46 ± 0.79 | - | - | - | - |
| PA dimer monoglycoside 2 | 2021 | - | - | 5.31 ± 4.62 a | - | 13.66 ± 7.03 b | 0.25 ± 0.09 | 243.68 ± 51.34 c | - |
| | 2020 | - | - | 86.05 ± 30.15 | - | - | - | - | - |
| naringenin hexoside 1 | 2021 | - | 390.52 ± 290.05 b | - | - | - | - | - | 0.16 ± 0.02 a |
| | 2020 | - | 12.06 ± 7.14 | - | - | - | - | - | - |
| naringenin hexoside 2 | 2021 | - | 0.21 ± 0.10 a | - | - | - | - | 1.39 ± 1.02 | 0.23 ± 0.16 a |
| | 2020 | - | 0.91 ± 0.50 | - | - | - | - | - | - |
| naringenin hexoside 3 | 2021 | - | - | - | - | - | - | - | 0.24 ± 0.08 |
| | 2020 | - | - | - | - | - | - | - | - |
| naringenin hexoside 4 | 2021 | - | - | - | - | - | - | 0.15 ± 0.03 | - |
| | 2020 | - | - | - | - | - | - | - | - |
| naringenin hexoside 5 | 2021 | - | - | - | - | - | - | 0.79 ± 0.29 | - |
| | 2020 | - | - | - | - | - | - | - | - |
| **TOTAL** | **2021** | **3695.83 ± 3541.84 a** | **2145.76 ± 1490.84 c** | **2579.61 ± 1487.31 a** | **973.65 ± 572.06 b** | **3608.40 ± 1061.89 a** | **483.07 ± 294.17 a** | **12293.14 ± 3132.78 b** | **295.23 ± 122.32 a** |

| | | | | | | | | | | |
|---|---|---|---|---|---|---|---|---|---|---|
| | | **2020** | **5848.32 ± 2201.74 a** | **1119.43 ± 516.31 b** | **9358.54. ± 4929.68 b** | **795.11 ± 392.95 a** | **-** | **-** | **-** | **-** |
| | quercetin galloyl hexoside 1 | 2021 | 0.51 ± 0.42 | - | - | - | - | 2.04 ± 1.72 b | - | 0.22 ± 0.01 a |
| | | 2020 | 2.33 ± 0.65 | - | - | - | - | - | - | - |
| | quercetin galloyl hexoside 2 | 2021 | 0.13 ± 0.03 a | - | 0.83 ± 0.55 c | - | 0.31 ± 0.07 b | - | - | 0.36 ± 0.35 |
| | | 2020 | 0.71 ± 0.21 a | - | 3.14 ± 1.65 b | - | - | - | - | - |
| | quercetin-3-rutinoside | 2021 | 0.09 ± 0.01 a | 341.02 ± 264.32 c | 4.16 ± 2.63 c | 371.06 ± 184.03 c | - | 0.22 ± 0.01 a | 1.44 ± 1.36 b | 0.56 ± 0.55 b |
| | | 2020 | 0.77 ± 0.19 a | 12.43 ± 7.05 a | 14.06 ± 8.04 b | 23.65 ± 18.54 b | - | - | - | - |
| | quercetin-3-galactoside | 2021 | 3.24 ± 1.96 a | 5.06 ± 2.65 ab | 13.85 ± 9.01 c | 70.44 ± 30.51 c | 6.20 ± 2.12 b | 7.45 ± 2.68 b | 2.03 ± 0.56 a | 2.65 ± 1.68 a |
| | | 2020 | 11.63 ± 6.04 a | 67.53 ± 39.62 a | 12.33 ± 9.11 a | 21.04 ± 12.64 b | - | - | - | - |
| | quercetin-3-glucoside | 2021 | 3.65 ± 1.64 b | 2.48 ± 1.69 a | 6.17 ± 3.64 c | 39.62 ± 19.63 b | 1.92 ± 0.71 a | - | 2.68 ± 0.78 b | 2.04 ± 0.91 a |
| | | 2020 | 5.53 ± 3.52 a | 2.06 ± 1.04 a | 31.48 ± 19.65 b | 6.73 ± 5.53 b | - | - | - | - |
| | kaempferol hexoside 1 | 2021 | 0.13 ± 0.03 a | - | 0.38 ± 0.27 c | - | 0.11 ± 0.02 a | 1.30 ± 0.65 | 0.18 ± 0.04 b | - |
| | | 2020 | 0.24 ± 0.16 a | - | 2.36 ± 1.04 b | - | - | - | - | - |
| | kaempferol hexoside 2 | 2021 | - | 3.06 ± 1.53 ab | - | 4.05 ± 2.04 b | - | - | 0.45 ± 0.11 | 1.97 ± 0.78 a |
| | | 2020 | - | 4.11 ± 3.36 b | - | 2.05 ± 1.03 a | - | - | - | - |
| | kaempferol derivate | 2021 | - | - | - | - | - | - | 17.09 ± 8.03 | - |
| | | 2020 | - | - | - | - | - | 1.62 ± 1.03 | - | - |
| | phloretin pentosyl hexoside 1 | 2021 | 0.19 ± 0.01 a | - | 1.44 ± 0.85 b | - | 12.54 ± 7.01 d | - | 23.62 ± 5.87 c | - |
| | | 2020 | 0.28 ± 0.15 a | - | 34.05 ± 21.33 b | - | - | - | - | - |
| flavonols | phloretin pentosyl hexoside 2 | 2021 | - | - | 3.65 ± 2.01 a | - | 7.66 ± 1.45 b | - | - | - |
| | | 2020 | - | - | 13.22 ± 7.05 | - | - | - | - | - |
| | quercetin-3-glucuronide | 2021 | 5.04 ± 3.62 b | - | 2.53 ± 1.90 a | 8.66 ± 6.53 | 7.41 ± 0.95 c | - | 78.04 ± 12.49 d | - |
| | | 2020 | 9.01 ± 6.54 a | - | 12.83 ± 6.22 | 11.66 ± 9.45 a | - | - | - | - |
| | quercetin-3-arabinopyranoside | 2021 | 6.82 ± 5.53 a | 0.33 ± 0.20 a | - | 0.41 ± 0.18 b | - | - | 25.11 ± 4.03 b | 1.64 ± 0.02 c |
| | | 2020 | 8.56 ± 5.53 | 0.47 ± 0.29 a | - | 0.51 ± 0.38 a | - | - | - | - |
| | quercetin-3-arabinofuranoside | 2021 | 6.44 ± 4.02 c | - | 1.48 ± 0.87 b | 1.95 ± 0.57 | 0.13 ± 0.03 a | - | 2.04 ± 0.56 b | - |
| | | 2020 | 2.04 ± 0.94 a | 0.28 ± 0.09 a | 1.50 ± 0.86 a | 0.76 ± 0.45 b | - | - | - | - |
| | isorhamnetin-3-rhamnoside | 2021 | 1.52 ± 0.93 a | - | 3.65 ± 2.04 bc | 3.68 ± 1.64 c | 1.62 ± 0.56 a | - | 1.93 ± 0.01 b | - |
| | | 2020 | 9.13 ± 6.98 b | - | 22.34 ± 11.30 a | 1.04 ± 0.51 | - | - | - | - |
| | isorhamnetin-3-hexoside | 2021 | - | - | 3.08 ± 1.89 b | 0.67 ± 0.39 | - | - | 1.44 ± 0.86 a | - |
| | | 2020 | - | - | 1.90 ± 0.89 | 0.22 ± 0.09 | - | - | - | - |

| | | | | | | | | | | |
|---|---|---|---|---|---|---|---|---|---|---|
| | Isorhamnetin pentoside 1 | 2021 | - | - | 6.85 ± 5.04 b | 23.65 ± 12.79 b | 0.85 ± 0.14 a | - | - | 1.03 ± 0.35 a |
| | | 2020 | - | - | 9.11 ± 6.41 | 18.65 ± 4.62 | - | - | - | - |
| | Isorhamnetin pentoside 2 | 2021 | - | 3.49 ± 1.06 b | 2.45 ± 0.19 b | 1.04 ± 0.91 a | 9.04 ± 1.69 a | - | - | - |
| | | 2020 | - | 2.05 ± 1.42 | 82.65 ± 20.50 | - | - | - | - | - |
| | quercetin-3-rhamnoside | 2021 | 0.13 ± 0.04 a | 5.66 ± 3.64 | 3.05 ± 0.23 b | - | 0.65 ± 0.11 c | - | - | - |
| | | 2020 | 22.05 ± 12.63 b | 4.33 ± 3.06 | 6.61 ± 2.06 a | - | - | - | - | - |
| | quercetin-acetylhexoside | 2021 | 22.05 ± 18.06 d | - | 6.17 ± 4.81 c | - | 0.68 ± 0.14 b | - | 0.22 ± 0.03 a | - |
| | | 2020 | 2.09 ± 1.60 b | - | 6.45 ± 1.45 a | - | - | - | - | - |
| | quercetin galloyl pentoside 1 | 2021 | 12.08 ± 8.44 c | - | 4.46 ± 3.46 b | - | 0.45 ± 0.03 a | - | - | - |
| | | 2020 | 0.01 ± 0 a | - | 4.32 ± 1.06 b | - | - | - | - | - |
| | quercetin galloyl pentoside 2 | 2021 | - | - | 0.42 ± 0.28 | - | - | - | - | - |
| | | 2020 | - | - | 0.10 ± 0.03 | - | - | - | - | - |
| | quercetin galloyl pentoside 3 | 2021 | - | - | 0.25 ± 0.17 | - | - | - | - | - |
| | | 2020 | - | - | 0.41 ± 0.28 | - | - | - | - | - |
| | quercetin-3-xyloside | 2021 | 0.46 ± 0.35 a | 1.98 ± 0.98 a | 0.61 ± 0.45 b | 1.63 ± 1.08 a | 6.12 ± 3.02 d | - | 1.67 ± 0.85 c | 4.02 ± 1.84 b |
| | | 2020 | 2.49 ± 1.66 b | 2.67 ± 1.93 a | 14.42 ± 7.65 a | 3.62 ± 1.82 b | - | - | - | - |
| | **TOTAL** | **2021** | **57.48 ± 45.09 b** | **363.08 ± 276.07 b** | **65.48 ± 40.29 b** | **526.86 ± 260.30 c** | **55.69 ± 18.05 a** | **12.63 ± 6.09 a** | **157.94 ± 35.58 c** | **14.49 ± 6.49 a** |
| | | **2020** | **76.87 ± 46.80 a** | **95.93 ± 57.86 a** | **273.28 ± 126.58 b** | **89.93 ± 55.06 a** | **-** | **-** | **-** | **-** |
| flavones | apigenin derivative 1 | 2021 | 0.09 ± 0.01 a | 5.04 ± 2.06 | 3.04 ± 2.67 b | - | 0.56 ± 0.45 d | - | 0.12 ± 0.02 a | - |
| | | 2020 | 0.34 ± 0.11 a | 1.16 ± 0.03 | 3.06 ± 2.45 b | - | - | - | - | - |
| | apigenin derivative 2 | 2021 | 8.14 ± 1.05 b | - | 2.17 ± 1.05 a | - | 8.21 ± 0.37 b | - | - | - |
| | | 2020 | 8.09 ± 3.06 b | - | 5.98 ± 3.87 a | - | - | - | - | - |
| | **TOTAL** | **2021** | **8.23 ± 1.06 c** | **5.04 ± 2.06** | **5.21 ± 3.72 b** | **-** | **8.77 ± 0.82 d** | **-** | **0.12 ± 0.02 a** | **-** |
| | | **2020** | **8.43 ± 3.17 a** | **1.16 ± 0.03** | **9.04 ± 6.32 a** | **-** | **-** | **-** | **-** | **-** |
| DIHYDROCHAL CONE | phloridzin | 2021 | 0.78 ± 0.04 b | **-** | 0.51 ± 0.44 a | **-** | **-** | 10.09 ± 4.38 a | - | 1098.45 ± 544.72 b |
| | | 2020 | 58.64 ± 36.02 a | **-** | 47.02 ± 13.11 a | **-** | **-** | - | - | - |

Note: (-) Compound was not detected.
